# Avrami Kinetics of Cylindrical Growth Under Hard-Wall Confinement: A Monte Carlo Study of Thin-Film Crystallization

**DOI:** 10.3390/polym18070840

**Published:** 2026-03-30

**Authors:** Catalin Berlic

**Affiliations:** Faculty of Physics, University of Bucharest, 405 Atomistilor Street, 077125 Magurele, Romania; cataliniulian.berlic@g.unibuc.ro

**Keywords:** polymer crystallization, thin-film confinement, Avrami kinetics, Monte Carlo simulation, cylindrical growth, geometric truncation, segmented regression

## Abstract

The Johnson–Mehl–Avrami–Kolmogorov (JMAK) formalism provides a classical framework for describing polymer crystallization kinetics; its applicability under finite-domain confinement requires quantitative assessment. In this work, the influence of one-dimensional geometric restriction on cylindrical growth in polymer thin films is investigated using a stochastic Monte Carlo approach. The model considers site-saturated nucleation on randomly distributed cylindrical nanofibers with constant radial growth velocity under hard-wall boundary conditions. Crystallization kinetics were evaluated through automated segmented regression of the double-logarithmic JMAK representation. Under confinement, the Avrami plot departs from single-slope linearity and exhibits two successive quasi-linear regimes characterized by effective parameter pairs $\left(n_{1},\ln k_{1}\right)$ and $\left(n_{2},\ln k_{2}\right)$. The primary exponent $n_{1}$ remains thickness-independent, consistent with early-stage radial expansion prior to boundary interaction. The secondary exponent $n_{2}$ displays a non-monotonic dependence on reduced film thickness, reflecting the competing influence of wall-induced truncation and inter-domain impingement on late-stage transformation. These results support a geometric interpretation in which finite-domain constraints modify the apparent Avrami response through the competing effects of wall-induced truncation and inter-domain impingement and provide a reproducible framework for analyzing dual-regime Avrami behavior in confined crystallization systems.

## 1. Introduction

The crystallization of polymers is a phase transition that dictates the morphological architecture and macroscopic performance, such as the thermal, mechanical, and barrier properties, of semicrystalline polymeric materials [1]. The macroscopic evolution of this phase transformation, from an amorphous melt to a crystalline solid, is classically formulated using the Johnson–Mehl–Avrami–Kolmogorov (JMAK) theory [1,2,3]. The theoretical foundation established by Avrami rests on the probabilistic concept of “extended volume”, which accounts for domain overlap and phantom nucleation events during both isothermal and non-isothermal crystallization processes [4]. This framework describes the hypothetical unrestricted growth of crystalline domains, explicitly including “phantom” domains [5] originating from theoretical nucleation attempts in already solidified areas [6,7,8,9], which is subsequently corrected for statistical impingement (overlap) to yield the actual transformed volume fraction [6,7,8,9,10,11]. While this analytical framework is highly successful for bulk, isotropic, three-dimensional systems, its predictive accuracy diminishes in the presence of complex geometries, such as directional nucleating agents, instantaneous growth processes, or severe spatial confinement [2,9], as topological constraints inherently violate the Avrami assumptions of constant nucleation and unrestricted growth rates [12].

In polymer nanocomposites and fiber-reinforced systems, massive surface nucleation triggers transcrystallization, leading to profound topological modifications in crystallization kinetics [11,13,14], where the increased density of heterogeneous nucleation on the fiber surface hinders lateral extension and forces directional growth. High aspect ratio additives, such as carbon-based nanofibers or nanosticks, act as highly active heterogeneous nucleation sites [15,16,17]. According to the classical theory of heterogeneous nucleation, the presence of foreign surfaces significantly lowers the critical free energy barrier by providing an interface that reduces the surface energy penalty required for the formation of a critical nucleus, often conceptualized through a contact angle or spherical cap model [5,11,18]. This energetic advantage leads to a massive, quasi-instantaneous surface-induced nucleation [4]. When this heterogeneous nucleation occurs with sufficiently high density along the interphase region, the lateral extension of the growing spherulites is severely hindered almost immediately by mutual impingement. Consequently, crystal growth is forced to proceed exclusively in one direction perpendicular to the fiber surfaces, developing a columnar layer known as transcrystallinity [13,19]. Thus, classical three-dimensional spherulitic growth is transformed into a predominantly one- or two-dimensional cylindrical (radial) growth mechanism [20].

The complexity of this process is amplified when the polymer matrix is simultaneously subjected to strict one-dimensional geometric confinement, such as in thin films bounded by hard walls [21,22,23]. Confinement significantly alters the thermodynamics and kinetics of crystallization, perturbing the free energy of the polymer and often severely reducing the overall crystallization rate, strongly dependent on the film thickness [21,23]. This slowing down is attributed to finite-size effects that limit the probability of finding active nuclei and to sluggish mass transport caused by irreversible chain adsorption at the impenetrable interfaces, which heavily restricts segmental mobility, increases the local effective viscosity, and raises the entropic barrier for stem formation [24,25,26,27].

Extensive literature reveals that reducing the spatial dimensionality and volume of heterogeneity-free microdomains forces the system to crystallize at extreme supercoolings, shifting the overall kinetics towards a first-order regime n≈1 governed almost exclusively by the surface or homogeneous nucleation step [28,29,30].

Furthermore, at the interface with impenetrable hard walls, the expanding crystalline volume is physically truncated. This boundary effect fundamentally violates the unbounded growth assumption of the standard JMAK extended volume. As detailed in comprehensive probabilistic models, when a sample has a finite thickness, nucleation sites beyond the geometrical boundaries are inherently absent. Because of this, the phantom overgrowth concept fails, and the geometric evaluation of the extended volume must mathematically account for the truncation of spherical or cylindrical caps by the material’s surfaces to avoid predicting an overgrowth that cannot physically exist [2,11,31].

Once a growing front intersects a hard physical boundary, a defined volume fraction of the theoretical envelope becomes geometrically inaccessible [10]. This, in turn, necessitates rigorous finite-domain corrections and finite-size scaling to describe the transformation rate accurately, because geometric truncation lowers effective transformation rates and skews the classical Avrami parameters [10,32,33,34,35].

Given the analytical difficulties in solving impingement and boundary truncation equations for such highly asymmetric topographies, stochastic Monte Carlo (MC) and Molecular Dynamics simulations have emerged as indispensable tools [26,36,37]. These computational algorithms can precisely capture the spatial evolution of the crystalline fronts, the interaction with physical nucleants, and the exact geometric impingement against confining boundaries [20,38]. Moreover, MC numerical integration methods provide a highly robust statistical approach to compute volume fractions and accessible phase domains in complex discrete environments without relying on simplified analytical formulas [39].

Building on this methodological framework, the present work investigates the Avrami kinetics of cylindrical growth, complementing recent MC simulations that have successfully captured the influence of high-aspect-ratio nanofillers on early-stage structural ordering in polymer nanocomposites [20]. The crystallization is initiated simultaneously by a random distribution of active nanofibers embedded in a polymer matrix, which is strictly confined within a thin film geometry (hard-wall confinement). Our primary objective is to isolate the effect of film thickness on the kinetic parameters. As demonstrated by the double-logarithmic JMAK representations derived from our simulation data, the transformation does not follow a single linear trend. Instead, the geometric truncation of the cylindrical fronts by the rigid boundaries fractures the Avrami behavior into two distinct and successive kinetic regimes [40].

We processed the simulation data by implementing an automated segmented regression and change-point detection algorithm [41,42]. In the Avrami representation, the procedure finds two approximately linear regimes, each associated with an effective growing exponent. The primary regime is characterized by the exponent $n_{1}$ which remains stable (within regression uncertainty) over a large range of film thicknesses, reflecting an initial phase of unhindered cylindrical radial growth. By contrast, the secondary regime exponent, $n_{2}$, exhibits a non-monotonic dependence on reduced thickness, governed by the competition between wall-induced truncation and collective volume impingement. From the intersection of the two fits, we extract the crossover time $t_{c}$ and the corresponding transformed fraction $X_{c}$, which provide a quantitative characterization of the crossover between the two effective kinetic regimes. Within the present geometric Monte Carlo framework, the observed dual-regime behavior is interpreted in terms of the gradual competition between wall-induced truncation and inter-domain impingement, rather than as a sharp dimensional transition.

## 2. Models and Methods

### 2.1. Simulation Framework and Geometry

The crystallization kinetics of a polymeric film in the presence of nanofibers as nucleation sites was investigated using a stochastic MC algorithm specially developed.

The simulation domain consists of a rectangular confinement box of dimensions L×L×H. To simulate a thin-film environment, the lateral dimensions were kept fixed while the thickness H was systematically varied, thereby isolating the effect of one-dimensional geometric confinement, as commonly adopted in studies of confined polymer crystallization [2,26,32,43]. Periodic boundary conditions were enforced along the OX and OY axes to emulate a semi-infinite planar medium and eliminate edge effects.

At the initial moment, the system contains a number of cylindrical fibers that act as instantaneous nucleation sites for a polymer crystalline phase, all geometrically confined within a thin film of height H. The simulated polymeric system is initially entirely amorphous and undergoes radial (cylindrical) crystallization using the pre-existing fibers as heterogeneous nucleation sites.

To ensure the universality of the results, all geometric parameters and growth velocity of the crystallites are expressed in reduced (dimensionless) units relative to the fiber diameter, $d_{\mathrm{fiber}}$. The fundamental spatial parameter is then the reduced thickness of the film, defined as follows:

(1) $$\tilde{H}=H/d_{\mathrm{fiber}}$$

In this study, the reduced lateral dimensions of the simulation box were fixed at $\tilde{L}=L/d_{\mathrm{fiber}}=1500$, derived from the absolute value of the side of the simulation box L=300 (arbitrary units) and $d_{\mathrm{fiber}}=2R_{\mathrm{fiber}}=0.2$ (arbitrary units).

### 2.2. Fiber Matrix Generation via Rejection Sampling

The fiber matrix serves as the fundamental scaffolding for the phase transformation, mimicking the behavior of a reinforced polymer composite in an ultra-dilute regime, as in [11]. The fibers are modeled as rigid, impenetrable cylinders of radius $R_{\mathrm{fiber}}$ that act as the exclusive heterogeneous nucleation sites for the polymer matrix, following the hypothesis applied to polymers in the vicinity of a curved impenetrable interface in [38].

The spatial distribution and orientation of these fibers are governed by a rejection sampling protocol:

- Fiber Geometry: Fibers are treated as rigid cylinders with a constant radius $R_{\mathrm{fiber}}=0.1$.
- Stochastic Placement: Each fiber is defined by a center randomly placed in the simulation box and a random orientation vector in three-dimensional space.
- Hard-Wall Condition (Strict Confinement): A generated fiber is accepted only if its entire segment, from entry to exit point, remains strictly within the vertical interval [0,H] (Rejection Sampling method). In other words, any fiber intersecting the planes Z=0 or Z=H is rejected. This mechanism ensures that the orientation distribution near the walls is modified naturally, favoring orientations parallel to the boundaries instead of simply reducing local density.
- Non-Exclusive Placement (Phantom Fibers): Consistent with the stochastic nature of the algorithm, fibers are allowed to intersect and overlap without physical penalty [1,8]. This approximation is justified by the dilute regime investigated here, corresponding to a volume fraction φ=0.002.

In a dilute random dispersion of objects, geometric intersections are binary events involving pairs of independently positioned fibers. The expected number of intersections per fiber scales with the number density ρ, which is proportional to the volume fraction φ. Consequently, the total fraction of volume affected by fiber–fiber overlaps scales as $\varphi^{2}$, i.e., as a second-order term in the dilute expansion.

For φ=0.002, this second-order contribution is of order $10^{-6}$, rendering the spatial fraction influenced by mutual intersections statistically negligible compared to first-order effects such as confinement by rigid boundaries. Therefore, inter-fiber impingement is expected to be minimal relative to the dominant wall-induced geometric truncation, and allowing geometric overlap represents a statistically consistent simplification in the present dilute regime.

At the beginning of the run, the system is stochastically populated with fibers by placing them one by one within the simulation box, a process subjected to the rejection sampling protocol against the film thickness constraints, until the target volume fraction is achieved.

The volume fraction of the fibers is calculated using a Point-in-Volume MC numerical integration method [37,39,44]. In our model, fibers are allowed to overlap, the algorithm used to calculate the volume fraction does not rely on a direct analytical formula, but rather on a statistical approach.

The calculation process is as follows: the program generates a fixed number of random test points, $N_{\mathrm{MC}}\;=\;100,000$, within the entire volume of the simulation box. For each test point, the algorithm calculates the shortest reduced squared distance ${\tilde{d}}^{2}$ to the axis of the nearest fiber. If ${\tilde{d}}^{2}\leq {\tilde{R}}_{\mathrm{fiber}}^{2}$, the point is classified as belonging to the “fiber” phase.

The volume fraction φ is determined as the ratio between the number of points that “hit” a fiber $N_{\mathrm{fiber}}$ and the total number of sampling points, $N_{\mathrm{MC}}$:

(2) $$\varphi =N_{\mathrm{fiber}}/N_{\mathrm{MC}}$$

To ensure this calculation remains consistent with the infinite lateral nature of the film, the MC calculation of the fibers’ volume employs periodic boundary conditions via the minimum image convention [45,46]. The minimum image convention is applied exclusively along the X and Y axes to simulate a continuous planar medium, while the Z axis remains non-periodic to enforce the physical confinement of the film thickness. This allows the algorithm to accurately determine the shortest distance between a test point and the fiber segments even when they cross the lateral boundaries, effectively treating the simulation box as a domain that is infinite in-plane but strictly bounded vertically.

This mechanism is implemented in such a way that generated points are checked sequentially against all fibers accepted through the rejection sampling protocol. Thus, the 0.2% volume fraction mentioned earlier represents the statistical target the algorithm aims for when populating the matrix. Using this procedure, by the end of fiber placement in the simulation box, the total number of fibers, $N_{\mathrm{fiber}}$ is also obtained. Accepted fibers are treated as rigid infinite cylinders with reduced diameter ${\tilde{d}}_{\mathrm{fiber}}=1$.

### 2.3. The Crystallization Mechanism

After the first stage of fiber generation within the simulation box, polymer crystallization begins. The transition of polymer from an amorphous melt to a crystalline state is governed by a surface-initiated growth process [3].

The model assumes that at time t=0, the entire surface of every accepted fiber becomes active and induces crystallization simultaneously. No induction time or sporadic appearance of new nuclei occurs. The crystal phase propagates outward from the fiber axis in an isotropic radial fashion. Growth occurs with a constant reduced velocity ${\tilde{v}}_{\mathrm{growth}}=0.5$ per time step. All the simulations were performed at this velocity.

The growth velocity of such magnitude is well-suited for a simulation box of $\tilde{L}\;=\;1500$ and the dilute regime φ = 0.002 because it ensures a high temporal resolution. The low ratio of growth velocity to box size ensures a fine-grained capture of the crystallization kinetics, allowing for the precise identification of the moment when the crystalline front meets the film thickness boundaries. On the other hand, the slow expansion ensures that the geometric truncation of crystalline regions by the rigid walls occurs well before any significant inter-domain impingement, effectively isolating the impact of spatial frustration on the Avrami exponents. Also, the velocity of 0.5 minimizes the change in transformed volume between consecutive time steps, ensuring that the MC test points can accurately track the growth without excessive statistical noise or discretization errors, providing numerical stability.

The crystal phase of the polymer propagates outward from the fiber axis in an isotropic radial fashion. At any given time t, the total reduced radius of the crystalline sheath surrounding a fiber is

(3) $$\tilde{R}\left(t\right)={\tilde{R}}_{\mathrm{fiber}}+{\tilde{v}}_{\mathrm{growth}}\cdot t$$

As these cylindrical fronts expand, they eventually encounter the rigid walls at Z=0 and $Z=\tilde{H}$. Since growth cannot penetrate these hard boundaries, the crystalline volume is truncated by these hard boundaries, as described in [11,32]. This geometric restriction takes into account the confinement [43], and causes the observed transition in the Avrami index from the primary regime $n_{1}$ to the secondary regime $n_{2}$.

Transformation progress is tracked using 100,000 MC test points randomly distributed within the simulation box. However, to ensure numerical convergence and minimize statistical noise in the highly confined regime (low $\tilde{H}$ values), the sampling density was increased to 500,000 points for films with $\tilde{H}<125$. This adaptive approach guarantees that the observed kinetic transitions and the calculated Avrami exponents are a direct consequence of the geometric constraints rather than numerical artifacts arising from a reduced sample volume, despite the reduced vertical dimension.

The phase identification mechanism consists of the classification of each MC test point P(x, y, z) in the simulation domain, analogous to the highly effective pixel coloring methods used in modern non-isothermal numerical models [32].

1. Fiber Core Identification (Hard Core Phase)

The algorithm first determines if a test point resides within the physical boundaries of the reinforcement. For each fiber i in the system, the minimum perpendicular relative distance ${\tilde{d}}_{i}$ to the fiber’s central axis is calculated. If ${\tilde{d}}_{i}<{\tilde{R}}_{\mathrm{fiber}}$, the point is classified as belonging to the fiber phase. These points are excluded from the polymer crystallization statistics as they represent the impenetrable volume of the reinforcement.

2. Crystalline Phase Determination (Growth Front)

If the point is not within a fiber, its state depends on the time-dependent expansion of the crystalline front. Since nucleation is considered instantaneous at the fiber-polymer interface, a test point is marked as crystalline if its relative distance to the nearest fiber axis satisfies ${\tilde{d}}_{i}<\tilde{R}\left(t\right)$, with $\tilde{R}\left(t\right)$ given from Equation (3), provided the point remains within the vertical boundaries of the film. This method inherently accounts for impingement: a point becomes crystalline as soon as it is reached by the first available growth front.

3. Amorphous Phase (Residual Volume)

Any test point that is neither inside a fiber nor reached by a crystalline growth front is classified as part of the amorphous phase. This represents the polymer melt that has not yet undergone the phase transition at time t.

Consistent with the volume fraction calculation, the evaluation of the relative distance ${\tilde{d}}_{i}$ for phase identification incorporates periodic boundary conditions via minimum image convention along the X and Y axes. This ensures that the crystalline growth front is continuous across the lateral boundaries of the simulation box.

The present model is formulated under a set of simplified kinetic assumptions, including constant radial growth velocity and instantaneous site-saturated nucleation at the fiber surface. These assumptions are introduced to isolate the geometric effects of confinement and to avoid additional coupling with temperature-dependent kinetics or interfacial molecular processes. In particular, molecular-scale effects such as chain entanglement and interfacial adsorption are not explicitly included. As a result, the simulations do not aim to reproduce the full physico-chemical complexity of polymer crystallization, but rather to capture the geometric effects of transformation under hard-wall confinement. Consequently, the extracted Avrami parameters should be interpreted within this framework, as effective descriptors of the kinetic response induced by geometric confinement rather than as intrinsic material properties. Accordingly, the present model is intended as a controlled geometric framework rather than as a direct quantitative representation of a specific experimental polymer-film system.

### 2.4. Data Analysis and Statistical Averaging

The analysis of crystallization kinetics requires a statistical framework to extract meaningful physical parameters from the raw simulation data. The processing pipeline was designed to handle the stochastic nature of the MC sampling and to objectively identify the transition between different growth regimes. To ensure statistical significance and minimize stochastic noise, especially critical in the dilute regime, 16 to 32 independent runs were performed for each reduced thickness height $\tilde{H}$. For each film thickness, the final kinetic parameters were obtained by averaging the results from these independent simulations.

The primary output of the simulation is the time-dependent crystalline fraction, X(t). To isolate the polymer kinetics from the reinforcement phase, the relative crystalline fraction was calculated as the ratio of test points marked as crystalline to the total number of points available in the polymer matrix:

(4) $$X\left(t\right)=\frac{N_{\mathrm{cryst}}\left(t\right)}{N_{\mathrm{MC}}-N_{\mathrm{fiber}}\left(t\right)}$$

$N_{\mathrm{MC}}$ is the total number of MC points thrown in the simulation box (100,000 or 500,000 depending on the film thickness), $N_{\mathrm{fiber}}\left(t\right)$ is the number of points that hit a fiber at time t and $N_{\mathrm{cryst}}\left(t\right)$ is the number of points that hit the crystalline phase at time t.

The kinetic analysis is based on the classical JMAK theory [6,7,8] which gives the volume of the crystalline fraction as a function of time:

(5) $$X(t)=1-\exp\left(-k\;t^{n}\right)$$

Here k is the kinetic rate constant, and n is the Avrami exponent related to nucleation and growth geometry [1,6,7,8]. In order to determine n and k from simulation data, Equation (5) is linearized utilizing the double-logarithmic transformation [1,5,6,37]:

(6) $$\ln\left[-\ln\left(1\;-X\left(t\right)\right)\right]=\;n\ln t\;+\ln k$$

However, due to well-documented controversies regarding the reliability of singular Avrami exponents in the presence of geometric truncation and transcrystallinity, singular linear fits are often insufficient [29]. Given the non-linearities introduced by spatial confinement, this relation is analyzed using a segmented regression approach to capture the shift in growth dimensionality as the front interacts with the film boundaries.

The linearization of the simulation data revealed that the crystallization process does not follow a single linear trend throughout the entire transformation. The data exhibit a gradual change in slope, leading to a deviation from strict linearity in the Avrami representation, as noted in [2]. This behavior indicates that the data cannot be described by a single linear model, and we therefore implemented an automated segmentation. This methodology relies on broken-line regression models to objectively estimate the unknown breakpoints connecting distinct kinetic regimes, ensuring a robust evaluation of the transition [41].

To eliminate subjectivity in identifying the separation between the two linear segments of the Avrami representation, an automated segmentation procedure was implemented. The optimization performs an exhaustive scan over all admissible separation points within the ordered Avrami-transformed data. Each candidate separation divides the dataset into an early-time segment and a late-time segment.

For each admissible separation, two independent ordinary least-squares regressions are computed on the respective subsets, yielding slopes, intercepts, and their standard errors. Candidate separations are filtered by imposing a relative uncertainty threshold on the extracted slopes, requiring the relative standard error of each slope to remain below 10%. Among the remaining admissible separations, the optimal transition point is selected by minimizing a combined objective function that balances the summed regression variances of the two segments with a mild penalty term that discourages excessively short intervals.

To ensure the robustness of the fit, a structural constraint $N_{\min}=6$ points was imposed on each segment, providing a sufficient number of data points to yield a reliable linear regression, preventing the algorithm from converging toward trivial solutions where a segment might appear perfectly linear due to an insufficient sample size [41,42].

Once the two optimal linear regimes are identified, the transition point between the two regimes is defined by their intersection of the linearized curves. Considering the two regression equations for each regime as $Y=n_{1}\ln t+\ln k_{1}$ and $Y=n_{2}\ln t+\ln k_{2}$, the transition time $t_{c}$ and the corresponding crystalline fraction $X_{c}$ are determined as follows:

(7) $$t_{c}=\exp\left(\frac{\ln\;k_{1}-\ln\;k_{2}}{n_{2}-n_{1}}\right)$$

(8) $$X_{c}=1-\exp\left[-\exp\left(n_{1}\;\ln\;t_{c}+\ln k_{1}\right)\right]\;$$

The uncertainties for these derived quantities were determined using the Gauss method of error propagation [47]. The total uncertainty in $t_{c}$ and $X_{c}$ accounts for the standard errors of the slopes and intercepts of the two lines obtained from the ordinary least-squares fit of each run.

After processing all 16–32 runs for a given film thickness H, ensemble averages were computed for each extracted parameter $\left(n_{1},\ln k_{1},n_{2},\ln k_{2},t_{c},X_{c}\right)$. The associated uncertainties were obtained by propagating the per-run regression errors under the assumption of uncorrelated measurements, following standard procedures of error analysis [47].

The reported uncertainties primarily reflect statistical variability associated with Monte Carlo sampling and segmented regression, where the Avrami parameters are obtained from least-squares linear fits of the Avrami-transformed data (Y=ln[−ln(1−X)] as a function of lnt), and their standard errors are derived from the residual variance of the fits. These uncertainties are further propagated in the calculation of derived quantities, such as the crossover time and conversion fraction, and averaged over independent simulation runs.

The stability of the extracted parameters, including the segmentation breakpoint, was assessed across independent simulation runs, which consistently showed the identification of two kinetic regimes despite minor stochastic fluctuations. It should be noted, however, that systematic uncertainties are not quantitatively evaluated separately within the present framework.

In particular, systematic limitations arise from the simplified kinetic assumptions of the model, including the instantaneous activation of nucleation sites at the fiber surface, the absence of induction time, and the assumption of constant growth velocity.

The extracted values $\left(n_{1},\ln k_{1},n_{2},\ln k_{2},t_{c},X_{c}\right)$ may retain some dependence on the specific segmented-regression procedure, including the exhaustive scan over admissible breakpoint positions, the minimum number of points per segment, the relative-error filtering of candidate fits, and the penalty term used in the breakpoint-selection criterion. These aspects should therefore be taken into account when interpreting the numerical values of the fitted parameters and the derived crossover quantities.

## 3. Results

### 3.1. Avrami Representation and Identification of Kinetic Regimes

**Figure 1:**
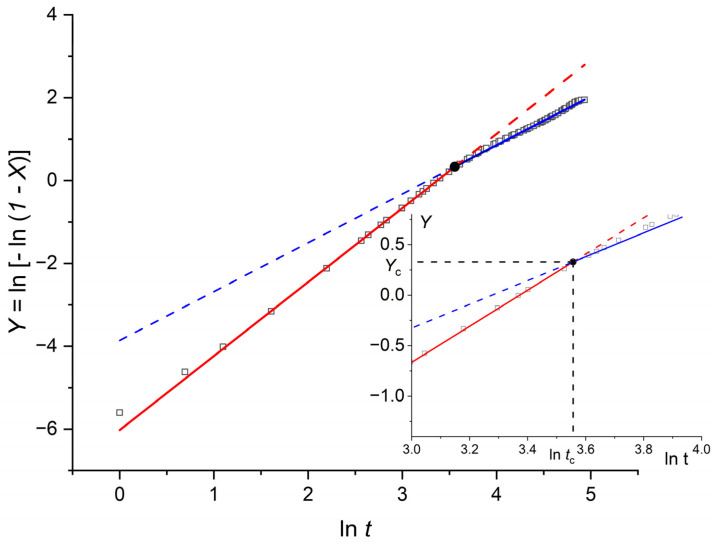
Avrami plot, $Y=\ln\left[-\ln\left(1-X\right)\right]$, as a function of lnt  for $\tilde{H}=400$. Simulation results are shown as open black squares; for readability, only 50% of the data points are displayed in the scatter plot (the underlying dataset is unchanged). Solid red and blue lines indicate the linear fits for the early- and late-time regimes, respectively, whereas the dashed segments show their extrapolated extensions for visual completeness. The marked intersection defines the crossover coordinates $\left(\ln t_{c},Y_{c}\right)$. The inset enlarges the crossover region, and dashed projection lines indicate the corresponding axis values.

The crystallization kinetics were analyzed using the Avrami representation, in which the double-logarithmic transformation

(9) $$Y=\ln\left[-\ln\left(1-X\right)\right]$$

is plotted as a function of lnt [1,6,7,8,48]. A representative example for $\tilde{H}=400$, corresponding to an intermediate thickness, is shown in Figure 1.

### 3.2. Evolution of the Avrami Exponents with Reduced Thickness

**Figure 2:**
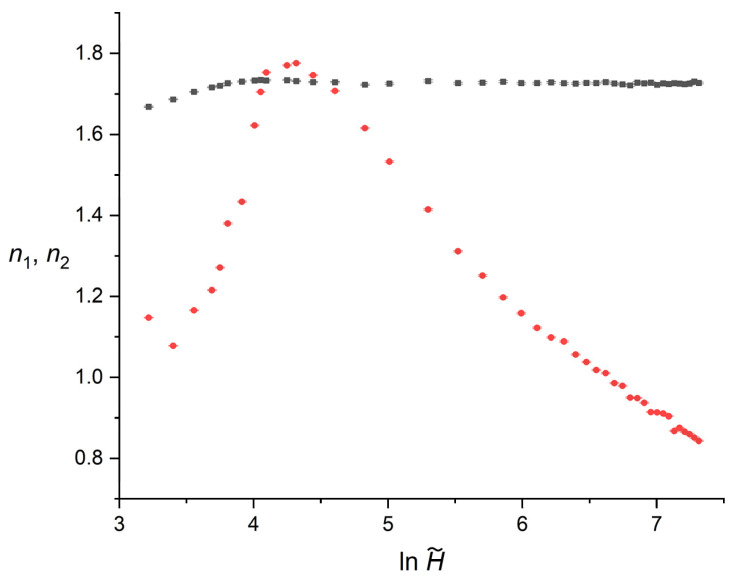
Dependence of the Avrami exponents $n_{1}$ (black squares) and $n_{2}$ (red circles) on the reduced thickness $\tilde{H}$. The horizontal axis is plotted on a logarithmic scale. Error bars represent standard deviations and are smaller than the symbol size.

Figure 2 illustrates the variation of the primary, $n_{1}$, and secondary, $n_{2}$, Avrami exponents as a function of the reduced film thickness. To properly resolve the behavioral shifts across the wide simulated domain, the horizontal axis is plotted on a logarithmic scale. Based on the extracted numerical data, the statistical uncertainties associated with both $n_{1}$ and $n_{2}$ are small, typically in the order of $10^{-3}$. Consequently, the error bars representing the standard deviations are smaller than the plotted symbols across the entire domain, indicating a very good precision in the determination of the individual kinetic slopes.

The primary exponent, $n_{1}$ (plotted as black squares in Figure 2), which dictates the early stage of crystallization, exhibits a highly stable behavior. At the lowest simulated thickness, $\tilde{H}=25$, $n_{1}$ registers a value of 1.668. It undergoes a negligible initial increase and immediately plateaus, maintaining a constant average value of approximately 1.73 throughout the remainder of the investigated thickness range, showing no systematic dependence on the spatial boundaries within the investigated thickness range, as stated in [49].

In contrast, the secondary exponent, $n_{2}$ (plotted as red circles in Figure 2), characterizes the late stage of crystallization, and displays a non-monotonic dependence on the confinement degree. Under extreme spatial restriction $\tilde{H}<30$, $n_{2}$ exhibits its lowest values, ranging between 1.078 and 1.147. As the thickness increases, the secondary exponent suffers an abrupt ascent, reaching a pronounced maximum of 1.776 at $\tilde{H}=75$ within the investigated thickness range. Notably, at this specific maximum point, $n_{2}$ slightly exceeds the stable baseline of the primary exponent $n_{1}=1.731$.

Following this peak, the system enters an intermediate transition zone where $n_{2}$ begins to decrease. As the thickness increases further beyond $\tilde{H}\approx 150$, the system stabilizes into the asymptotic thick-film regime. Across this extensive upper range of the simulated domain, the secondary exponent diverges significantly from $n_{1}$, ultimately dropping to 0.843 at the maximum investigated thickness $\tilde{H}=1500$, where the crystallization kinetics are no longer governed by wall-induced confinement but by inter-domain impingement.

The simulations were limited to a maximum reduced thickness of $\tilde{H}=1500$, a value equal to the lateral dimensions of the simulation cell, $\tilde{L}=1500$. Given the application of periodic boundary conditions along the x and y axes, maintaining $\tilde{H}\leq \tilde{L}$ ensures that the spatial development of the transcrystalline layers remains physically consistent with a film geometry. Increasing the thickness beyond this symmetry point would result in a disproportionate aspect ratio where the vertical growth path exceeds the lateral periodicity, potentially introducing finite-size artifacts that could obscure the characteristic features of the asymptotic thick-film regime.

### 3.3. Evolution of the Crossover Time with Reduced Thickness

**Figure 3:**
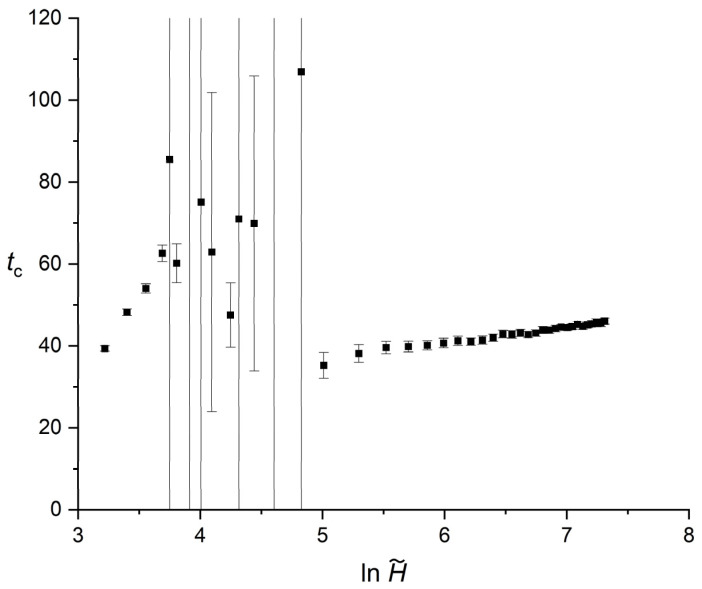
Dependence of $t_{c}$ on the reduced thickness $\tilde{H}$ (horizontal axis plotted in logarithmic scale). Error bars represent standard deviations.

Figure 3 illustrates the evolution of the crossover time $t_{c}$ as a function of the reduced film thickness. The horizontal axis is presented on a logarithmic scale. The results reveal three distinct stability regimes that are fundamentally linked to the convergence or divergence of the primary and secondary Avrami exponents.

In the extreme confinement regime $\tilde{H}\leq 35$, $t_{c}$ exhibits a stable, monotonic increase, starting from 39.36. In this region, the high contrast between $n_{1}$ and $n_{2}$ ensures a mathematically well-defined intersection, resulting in minimal statistical uncertainty.

As the system enters the intermediate transition zone $35\leq \tilde{H}\leq 125$, where $n_{1}$ and $n_{2}$ approach each other, the geometric determination of the crossover point becomes progressively ill-conditioned. Since $t_{c}$ is defined as the intersection of two nearly parallel linear segments in the Avrami representation; small statistical variations in the slopes or intercepts produce amplified variations in the intersection coordinate, see Equation (7) [42]. At $\tilde{H}=57.5$, the regression procedure formally returns an unbounded value of $t_{c}$, as the difference $n_{1}-n_{2}$ approaches zero in Equation (7). This behavior reflects the mathematical structure of the intersection formula rather than a physical divergence of the crystallization kinetics. For clarity of presentation, these ill-conditioned cases are omitted from Figure 3, as their inclusion would compress the scale of the well-defined regimes. The large error bars observed in this interval therefore represent amplified geometric uncertainty associated with nearly parallel fits, not an intrinsic instability of the transformation process.

Finally, as the thickness increases beyond $\tilde{H}\geq 150$, the system enters the asymptotic thick-film regime. In this domain, the crossover time recovers its stability and follows an approximately monotonic increasing trend, rising from 35.24 to 46.04 at $\tilde{H}=1500$. Across this entire range, the statistical uncertainties become small, with standard deviations below 1.0, consistent with the improved conditioning of the two-segment intersection once the Avrami exponents are sufficiently separated.

### 3.4. Evolution of the Critical Transformed Fraction

**Figure 4:**
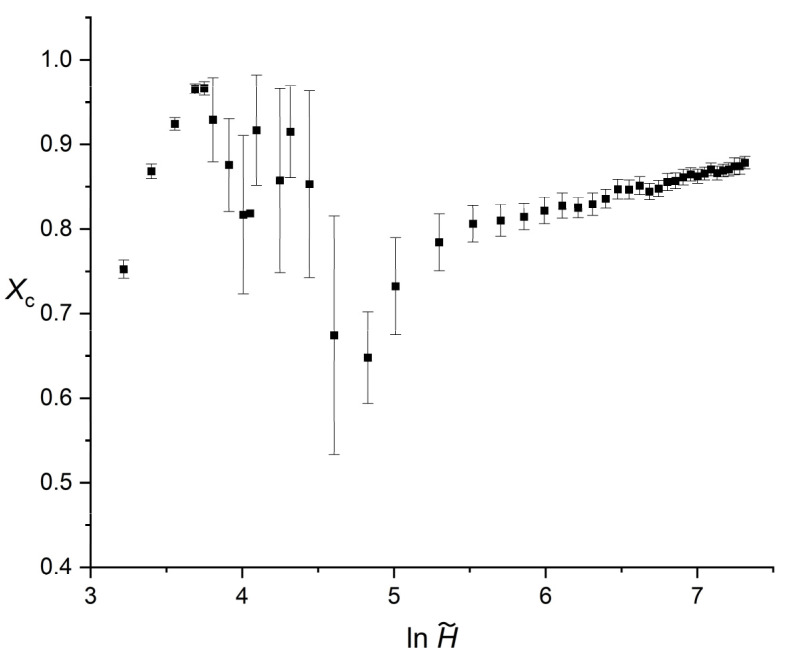
Dependence of $X_{c}$ on the reduced thickness $\tilde{H}$ (horizontal axis plotted in logarithmic scale). Error bars represent standard deviations.

Figure 4 presents the evolution of the critical transformed fraction $X_{c}$, representing the degree of crystallinity at the crossover point, as a function of the reduced film thickness. Consistent with the previous kinetic parameters, the horizontal axis is plotted on a logarithmic scale. The overall trend of $X_{c}$ follows a complex, non-monotonic profile that reflects the shifting dominance between primary and secondary crystallization regimes.

In the extreme confinement regime, $\tilde{H}\leq 35$, the transformed $X_{c}$ exhibits a rapid and stable increase. Starting from a value of 0.752 at $\tilde{H}=25$, it reaches a local maximum of 0.966 at $\tilde{H}=42.5$. In this region, the statistical uncertainties are remarkably low, with standard deviations consistently below 0.01. This indicates that under strict spatial restriction, the crossover point occurs very late in the process, meaning the primary crystallization stage accounts for nearly the entire volume transformation before the onset of wall-induced impingement.

As the system enters the intermediate transition zone, $35\leq \tilde{H}\leq 125$, the behavior of $X_{c}$ becomes highly unpredictable. The values drop sharply from the previous peak, reaching a minimum of approximately 0.648 at $\tilde{H}=125$ within the investigated thickness range. Similar to the crossover time $t_{c}$, this region is characterized by significantly larger error bars (reaching a standard deviation of 0.141 at $\tilde{H}=100$). The increased statistical scatter is a direct consequence of the fact that these crossover parameters are determined from Equation (7), having in the denominator the difference $\left|n_{1}-n_{2}\right|$. As the primary $n_{1}$ and secondary $n_{2}$ exponents approach each other, the sensitivity of the intersection point to minor numerical fluctuations increases, leading to less reliable determinations of the critical fraction.

Finally, upon entering the asymptotic thick-film regime, $\tilde{H}\geq 150$, the system recovers its numerical stability. In this domain, $X_{c}$ exhibits a steady, linear, and monotonic increase with thickness. From an initial value of 0.732 at $\tilde{H}=150$, the critical fraction gradually rises to 0.878 at the maximum investigated thickness of $\tilde{H}=1500$. In this stable regime, the error bars become small almost completely, with standard deviations returning to values below 0.01 (e.g., $\tilde{H}=1500$), confirming that the crossover between early-stage growth and inter-domain impingement becomes once again a well-conditioned geometric intersection between the two fitted regimes.

## 4. Discussion

### 4.1. Validation of the Kinetic Framework in the Bulk Limit

Before addressing confinement effects, the kinetic framework was evaluated in the absence of geometrical constraints. A reference bulk simulation was performed using the same growth velocity, fiber volume fraction, stochastic fiber distribution, MC sampling density, and segmented fitting procedure as in the confined-film simulations. The only modification consisted of removing the vertical confinement boundaries.

**Figure 5:**
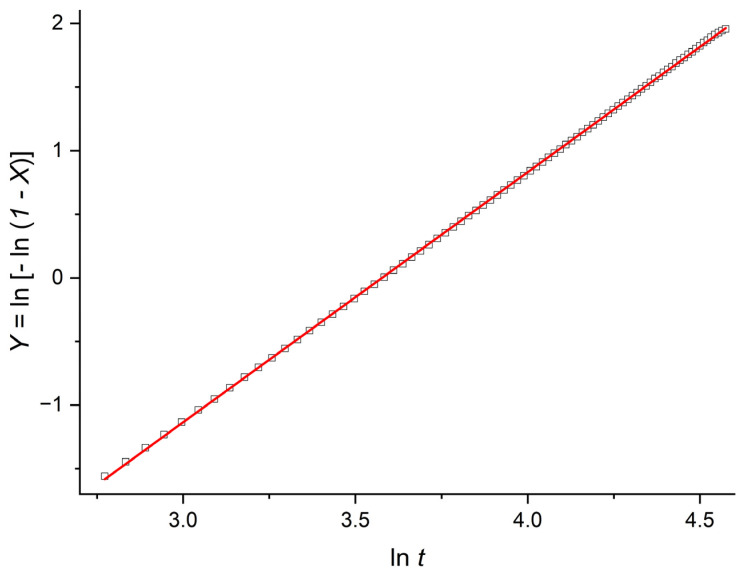
Avrami representation Y versus lnt for the bulk reference simulation performed without geometrical confinement. The solid line represents the linear fit.

The corresponding double-logarithmic Avrami representation Y plotted against lnt is shown in Figure 5.

The double-logarithmic representation displays a uniform linear behavior over the analyzed interval, consistent with a single kinetic regime.

The resulting Avrami analysis yielded $n_{\mathrm{bulk}}=1.9675\pm 0.0016$ with a coefficient of determination $R^{2}=0.99995$. This value is close to the theoretical expectation of n=2 for instantaneous (site-saturated) nucleation combined with cylindrical radial growth [1,5,6,7,8]. In this configuration, the extended transformed volume scales as $Y(t)\propto R(t{)}^{2}\propto t^{2}$, such that the effective dimensionality entering the Avrami exponent corresponds to that of the advancing radial front rather than to that of the embedding three-dimensional space, as derived within the JMAK framework [1,6,7,8].

The small deviation from the ideal value does not affect the qualitative dimensional interpretation of the growth process.

This bulk reference provides a baseline for interpreting deviations observed in finite films as consequences of geometrical confinement in a finite domain.

The emergence of the dual Avrami regime can be interpreted as a consequence of the competition between two geometrical constraints: wall-induced truncation of the growing cylindrical domains and inter-domain impingement [11,35,49]. In the early stage, the growth proceeds with limited domain impingement and negligible boundary effects, resulting in a stable primary exponent $n_{1}$. As the transformation advances, the progressive increase in domain impingement and the growing influence of boundary-induced truncation lead to a gradual modification of the effective growth kinetics [32,43,50]. In this framework, the transition between regimes should not be interpreted as a sharp physical event, but rather as a gradual change in the relative influence of domain impingement and boundary-induced truncation.

### 4.2. The Extreme Confinement Regime

The divergence between the primary $n_{1}\approx 1.7$ and secondary $n_{2}\approx 1.1$ Avrami exponents observed for $\tilde{H}<35$ reveals a fundamental shift in the crystallization geometry forced by the film boundaries. While the bulk reference n≈1.97 confirms an unhindered radial expansion of the transcrystalline layers, extreme confinement effectively cuts this three-dimensional development.

The stability of $n_{1}$ suggests that initial nucleation and early-stage growth remain largely unaffected by the walls, as the incipient crystalline lamellae have not yet reached the vertical boundaries. At the same time, the decrease of $n_{2}$ to values close to unity is consistent with a strongly confinement-modified late-stage Avrami response imposed by vertical confinement. In this regime, once the transcrystalline front impinges upon the z=0 and $z=\tilde{H}$ surfaces, the only available volume for further transformation lies in the xy-plane, between the fibers [28]. This geometric spatial confinement progressively modifies the late-stage kinetic response of the system, as the available space can no longer sustain the radial expansion characteristic of the bulk-like cylinders, an effect consistent with the analytical predictions of modified Avrami-Evans theories for volume-limited systems [4,35].

Furthermore, the very high critical fraction $X_{c}\approx 0.96$ found at these thicknesses shows that, under severe confinement, the primary growth stage is not merely a precursor, but the dominant mode of crystallization. The system effectively completes the vast majority of its phase transformation before the secondary, impingement-limited regime can even establish its kinetic signature. This suggests that in ultra-thin composite films, the “late-stage” crystallization is almost entirely suppressed, as the available amorphous space is exhausted nearly simultaneously with the first contact between the growing fronts and the impenetrable walls, as in [40].

While the present MC simulations capture the geometric truncation of expanding cylindrical fronts and predict a reduction of the secondary Avrami exponent toward values close to unity under strong confinement, $\tilde{H}<35$, these findings must be interpreted within the physical limitations of real multiphase polymer systems. The stochastic model isolates geometric effects by assuming constant radial growth velocity and unmodified thermodynamics up to the impenetrable boundaries.

Experimental studies on ultra-thin polymer films indicate that when the film thickness approaches nanometric scales, crystallization may become strongly suppressed rather than merely geometrically constrained [24,27]. This behavior has been associated with interfacial immobilization and the formation of low-mobility layers near solid substrates, which reduce segmental mobility and modify local transport properties.

Consequently, the reduction of the effective Avrami exponent predicted here should be regarded as the geometric limit of confinement within the assumptions of the model. In real nanocomposites, additional interfacial thermodynamic and kinetic effects are expected to interact with, and potentially dominate over, purely geometric truncation as the confinement length scale approaches that of immobilized interfacial layers.

However, experimental studies demonstrate that intrinsic crystal nucleation in isolated few-chain polymer droplets can remain unaffected by a high degree of spatial confinement [51]. This observation reinforces our premise, highlighting that the severe kinetic slowdowns isolated in our simulations stem fundamentally from volumetric and geometric factors, specifically, the physical truncation of the growing front, rather than from an alteration of the intrinsic thermodynamic nucleation barrier.

### 4.3. The Intermediate Transition Zone

The intermediate thickness range $35\leq \tilde{H}\leq 150$ represents a regime of intense kinetic competition. Unlike the extreme confinement regime, where boundary effects dominate the late-stage transformation, the increased spacing in this interval allows the simultaneous action of wall-induced truncation and inter-domain impingement, as described in [52].

This competition is reflected in the behavior of the secondary Avrami exponent, $n_{2}$. As $\tilde{H}$ increases toward 75, $n_{2}$ rises sharply and reaches a maximum value of 1.776, temporarily exceeding the primary exponent $n_{1}=1.731$. This behavior suggests a thickness interval in which wall-induced truncation and inter-domain impingement become comparable in influence. Accordingly, this interval is interpreted here not as a sharp dimensional crossover in the strict geometric sense, but as a competition regime in which the relative importance of these two constraints changes progressively during transformation.

The effective kinetic response in this thickness range should therefore be regarded as an apparent consequence of competing confinement effects within the Avrami representation, rather than as evidence of a distinct intrinsic growth mechanism or a new intrinsic growth dimensionality. This interpretation is consistent with recent literature showing that, under confinement, Avrami exponents are often treated as effective kinetic descriptors influenced by interfaces, nanoconfinement, and restricted growth volume rather than as direct measures of intrinsic growth dimensionality [4,26,50,53,54].

A further important feature of this regime is the loss of numerical stability in the crossover parameters because of the convergence of $n_{1}$ and $n_{2}$ toward nearly identical values. Since the crossover point $t_{c}$ is determined by the intersection of two power-law slopes, the near equality $n_{1}\approx n_{2}$ makes the intersection point hypersensitive to minor statistical fluctuations in the crystalline fraction.

### 4.4. The Asymptotic Thick-Film Regime

As the reduced thickness increases beyond the threshold, $\tilde{H}\geq 150$, the system enters the asymptotic thick-film regime, where the influence of the impenetrable vertical walls becomes negligible. This regime is characterized by a remarkable recovery of numerical stability, with the crossover parameters $t_{c}$ and $X_{c}$ exhibiting a linear dependence on thickness.

The main feature of this kinetic regime is the stability of the primary Avrami exponent $n_{1}$ together with a monotonic decrease of the secondary Avrami exponent $n_{2}$ over the thickness range considered. The exponent $n_{1}$ remains bulk-like, staying close to the value obtained from the independent unconfined reference simulation, $n_{\mathrm{bulk}}\approx 1.97$, consistent with an early-time growth stage that is only weakly affected by confinement. By contrast, in the large $\tilde{H}$ limit, the late-stage exponent $n_{2}$ drops significantly below unity, reaching 0.843 at $\tilde{H}=1500$.

This indicates that the dominant geometric constraint shifts from wall-induced truncation to inter-domain impingement. In these thick composite films, the late-stage crystallization is dominated by the collision and overlap of transcrystalline cylinders originating from neighboring fibers, as indicated in [11,13]. This process reduces the accessible amorphous volume to spatially constrained regions between neighboring fibers, resulting in effective Avrami exponents $n_{2}<1$, which are consistent with growth under strong impingement conditions.

The recovery of stability of parameters $t_{c}$ and $X_{c}$ (with standard deviations for $t_{c}$ dropping below 1.0) further confirms that the kinetic inversion is no longer a factor. In this asymptotic limit, the primary $n_{1}$ and $n_{2}$ exponents are sufficiently diverged, ensuring a mathematically well-defined and physically consistent crossover point, see Equation (7). The system thus stabilizes into a macroscopic composite state where the kinetics are dictated solely by the fiber distribution and the resulting impingement patterns, independent of the film’s total thickness.

The present analysis isolates the effect of film thickness by keeping the nucleation-site density, mean fiber spacing, and growth velocity fixed throughout the simulations. Among these parameters, the nucleation-site density and the corresponding mean fiber spacing are expected to influence the late-stage Avrami response by modifying the onset and relative importance of inter-domain impingement [19,20]. Within the present geometric framework, a change in constant growth velocity would be expected to affect the characteristic transformation times more directly than the geometric basis of the two effective regimes. Accordingly, the values of $n_{2}$, as well as the crossover descriptors $t_{c}$ and $X_{c}$, should be regarded as conditional on the specific parameter set adopted here. A systematic exploration of these additional control parameters would therefore remain a natural extension of the present framework.

Although the present implementation is restricted to cylindrical growth under hard-wall confinement, the combined simulation-and-segmented-regression framework could, in principle, be extended to other growth geometries and confinement conditions, provided that the resulting effective kinetic parameters are interpreted within the corresponding geometry-specific context.

Recent studies on confined polymer systems and related restricted geometries have shown that confinement and interfacial restrictions can modify crystallization kinetics, the Avrami response, and the effective growth geometry [19,25,55,56,57]. Similarly, recent simulation studies have highlighted the role of nanoconfinement and filler-induced geometric restrictions on crystallization behavior [20,26]. Compared with these experimental and numerical studies, which primarily report confinement-induced modifications of apparent crystallization kinetics and growth geometry, the present work addresses a more specific question, namely, how hard-wall confinement modifies the Avrami response of cylindrical growth initiated at line-like heterogeneities. In this context, the present study provides a systematic analysis of two successive effective Avrami regimes and the quantitative extraction of $n_{1}$, $n_{2}$, $t_{c}$, and $X_{c}$ over a broad range of reduced film thicknesses by means of automated segmented regression. The present results therefore do not introduce a new microscopic crystallization mechanism, but support a geometric interpretation in which early-stage growth and late-stage confinement-modified kinetics become distinguishable as two effective regimes under hard-wall restrictions.

## 5. Conclusions

This study shows that the crystallization kinetics of polymers under hard-wall confinement, when initiated by cylindrical growth fronts, exhibit a reproducible dual-regime behavior in the standard Avrami (JMAK) representation. Within the present geometric Monte Carlo framework, this behavior should not be interpreted as a sharp dimensional transition or as evidence of a distinct microscopic crystallization mechanism. Rather, it is consistent with a gradual redistribution of the relative influence of wall-induced truncation and inter-domain impingement during transformation, which progressively modifies the apparent Avrami response under confinement [2].

To analyze this behavior in a strictly operational and reproducible manner, we implemented an automated segmented-regression procedure. This approach yields two distinct Avrami parameter sets $\left(n_{1},\ln k_{1}\right)$ and $\left(n_{2},\ln k_{2}\right)$, defined by the slope-intercept pairs of successive linear segments.

Our findings reveal that the evolution of these parameters is governed by three distinct confinement regimes based on the reduced thickness $\tilde{H}$:

- Extreme Confinement, $\tilde{H}<35$: The secondary exponent is highly sensitive to confinement, approaching unity $\left(n_{2}\approx 1\right)$, consistent with a strongly confinement-modified late-stage Avrami response under strong wall-induced truncation, as discussed in [43]. This geometric restriction is also consistent with experimental findings in highly confined polymer nanodomains, where overall crystallization may approach first-order kinetics, n≤1, under nucleation-dominated conditions [28]. In this regime, the crossover descriptors $t_{c}$ and $X_{c}$ remain comparatively stable due to the large difference between the primary and secondary slopes.
- Intermediate Transition Zone, $35\leq \tilde{H}\leq 150$: In this interval, $n_{1}$ and $n_{2}$ attain comparable values. As a result, the geometric determination of the crossover point becomes ill-conditioned, and the derived quantities $t_{c}$ and $X_{c}$ exhibit amplified uncertainty due to the near-parallel character of the fitted segments. This interval should therefore be regarded as a competition regime rather than as a sharply defined transition.
- Asymptotic Thick-Film Regime, $\tilde{H}\geq 150$: Numerical stability is progressively recovered as the influence of confinement diminishes. However, the secondary exponent stabilizes at a sub-unitary $\left(n_{2}\approx 0.84\right)$, consistent with late-stage growth still being shaped by inter-domain impingement and finite-domain effects.

In summary, this work provides a workflow for extracting dual-regime Avrami parameters from MC simulations of thin-film crystallization under geometric confinement. This methodology allows a controlled separation between geometric effects and microscopic kinetic assumptions, enabling a consistent comparison of various confinement levels. The proposed framework is versatile and can be readily transferred to other confined growth geometries or boundary-condition models, provided the Avrami representation remains a suitable reduced description of the system.

## Data Availability

The original contributions presented in the study are included in the article, further inquiries can be directed to the author.

## Abbreviations

The following abbreviations are used in this manuscript:

JMAK	Johnson–Mehl–Avrami–Kolmogorov
MC	Monte Carlo
